# Anatomical peculiarities of dissection in the transabdominal preperitoneal procedure for inguinal hernias

**DOI:** 10.25122/jml-2023-0235

**Published:** 2023-06

**Authors:** Victor Dumitrescu, Laura Carina Tribus, Tiberiu Trotea, Daniel Ovidiu Costea, Dan Dumitrescu

**Affiliations:** 1 4^th^ Surgery Department, University Emergency Hospital, Carol Davila University of Medicine and Pharmacy, Bucharest, Romania; 2 2^nd^ Internal Medicine and Gastroenterology Department, Ilfov County Clinical Emergency Hospital, Carol Davila University of Medicine and Pharmacy, Bucharest, Romania; 3 Faculty of Medicine, Ovidius University, Constanta, Romania

**Keywords:** hernia, anatomical peculiarities, TAPP procedure

## Abstract

Inguinal hernia, a common surgical pathology, has substantial medical, social, and economic implications. Over time, various repair techniques have been explored to optimize outcomes, considering multiple postoperative factors beyond recurrence risk. This article aims to define anatomical and technical aspects impacting the immediate and late postoperative evolution of patients with inguinal hernia. Precise knowledge of anatomical structures and standardized surgical gestures result in the reduction of intraoperative and postoperative complications. Throughout history, the alloplastic procedure has demonstrated superiority over the anatomical approach, reinforcing the potential for ongoing advancements. Correct performance according to well-defined principles improves patients' quality of life after inguinal hernia surgery. These principles encompass the exact knowledge of anatomy, dissection steps, dissection limits, the sequence of dissection, and the prosthetic materials used. We describe our approach, with the laparoscopic method representing over 90% of cases at our clinic, indicating the shift towards minimally invasive techniques and emphasizing adherence to rigorous principles to achieve low perioperative complications.

## INTRODUCTION

The introduction of laparoscopy has brought about a paradigm shift in the surgical treatment of inguinal hernia, offering a new perspective on the anatomy of the inguinal region. With this change, the view of the groin region shifted from the well-established anterior-to-posterior approach to a posterior-to-anterior view, accompanied by multiple surgical approaches for hernia repair [1–5]. Although a new surgical technique has emerged, hernia repair principles have remained constant.

In 1878, Billroth mentioned, "If it is possible to artificially produce tissue with the density and resistance of a fascia or tendon, then we can discover the secret of the radical surgical cure of hernia"[1]. Despite the Nobel Prize-winning discovery of polypropylene by Karl Ziegler and Giulio Natta in 1963, Billroth's vision has not been fully realized [6]. This raises the question of why polypropylene alone is insufficient and introduces the need for novel anatomical and physical concepts to achieve better results and ultimately attain the radical surgical cure of inguinal hernia and beyond [7, 8].

Moreover, we have to consider the complexity of the human body and subsequent complications such as postoperative pain, migration of prosthetic material, iatrogenic injuries (intestinal perforations, bladder perforations), bleeding, osteitis, hematomas and seromas [9, 10]. Laparoscopic surgery is a relatively new field, but there is tremendous pressure to gain widespread acceptance, thus forcing young surgeons to perform surgeries without adequate proficiency, resulting in a high incidence of postoperative complications [11, 12]. Although laparoscopic inguinal hernia repair was first introduced over 27 years ago as an alternative to the open anterior approach for inguinal hernia repair, the majority of hernias worldwide are still repaired using the open anterior approach [13]. Despite numerous studies highlighting the post-operative benefits and safe applicability of laparoscopic inguinal hernia repair, its adoption has been stagnant. Although national societies such as the American Hernia Society and European Hernia Society have endorsed the technique, it has not gained the same popularity as other laparoscopic approaches. We have to wonder why so many young doctors have not adopted laparoscopic inguinal hernia repair as the procedure of choice [14]. By analyzing the clinical implications of hernia repair and correlating them with the anatomical concepts, we can provide a standardized approach to transabdominal preperitoneal repair (TAPP).

The International Collaboration Hernia Group introduced the operative concept of a Critical View of the Myopectineal Orifice (CVMO), defined as the "appropriate exposure of the anatomical area that must be attained before mesh placement". This new concept of CVMO has led to the standardization of surgical repair for inguinal hernia defects using TAPP and TEP procedures, establishing new approaches with the posterior view of the anatomy of the inguinal region (the five triangles and the "inverted Y") [9]. Surgical procedures using the CVMO concept are thought to reduce complications and improve understanding of surgical techniques.

In recent years, minimally invasive surgery has been gaining ground over the classic approach, taking into account the evolution of technology and the desire of the surgeon to perform at the highest level. Thus, new techniques for inguinal hernia repair (TAPP, TEP, R-TAPP) have also emerged. However, before referring to them, we would like to highlight the enormous potential brought by understanding the anatomy of the inguinal region seen from a different perspective: posterior to anterior [5, 15–17]. We would like to highlight some essential steps in the evolution of the surgical treatment of hernias in establishing a standardized procedure for faster recovery of the operated patient and the percentage reduction of postoperative complications. The present study describes the anatomy of the inguinal region, focusing on identifying the myopectineal orifice. Furthermore, we report on the standardized dissection performed in 127 surgeries conducted at our clinic. This article intends to help surgeons, promote a steeper learning curve, and reduce complications.

## BACKGROUND

### The anatomy of the inguinal region

Understanding the inguinal region anatomy is crucial for safe and effective hernia repair. Two key elements in this region are ligaments and fossae.

#### Ligaments

Ligaments play a crucial role in classifying hernias and guiding the dissection of the peritoneum to create an ideal flap. Two significant ligaments in the inguinal region are the median and lateral umbilical ligaments. The median umbilical ligament, a fibrous cord that connects the bladder to the umbilicus, forms itself as an allantoic stalk during fetal development and contains obliterated urachus. Its role in the laparoscopic approach to TAPP is to help classify inguinal hernia and determine the orientation of the optical telescope. Additionally, it allows for wide dissection in the surgical cure of bilateral inguinal hernias. The risk of increased bleeding in this ligament should also be mentioned, as it may contain an obliterated umbilical artery (in some cases, this is remnant). The lateral umbilical ligament contains the epigastric vessels (the epigastric artery is a branch of the external iliac artery). It plays an important role in delimiting the lateral space (Bogros) from the medial space (Retzius). At the same time, it helps determine the type of hernia (lateral/indirect or medial/direct) [9, 18].

#### Fossae

The posterior-to-anterior approach used to repair abdominal inguinal defects involves the lateral, medial, and bladder fossae. Each has its significance in identifying specific hernia types. The lateral fossa is found between the lateral umbilical ligament and the iliopubic tract, where the spermatic vessels and the deep inguinal ring meet. An indirect inguinal hernia may be found in this fossa. The medial fossa is bounded by the lateral umbilical ligament to the lateral and the medial umbilical ligament to the medial, and at this level, the direct hernia is highlighted. The bladder fossa is found medially to the medial and cranial umbilical ligament against the iliopubic tract. It can hide various types of hernia [9, 19–22]. Claus *et al*. describe that the three dissection zones for the surgical cure of the inguinal hernia TAPP procedure are established according to the three fossae bounded by the umbilical ligaments [6].

### Triangle of doom, triangle of pain, and corona mortis

Triangle of doom is a term originally used by Spaw in 1991 in relation to a region between the lateral gonadal vessels and the ductus deferens/medial round ligament [10]. It is located between the ductus deferens (medial) and the spermatic vessels (lateral). It is bordered inferiorly by the peritoneal fold and contains the external iliac vein with artery, the deep circumflex iliac vein, the genital branch of the genitofemoral nerve, and the femoral nerve. Injury to the elements of the triangle of doom could cause profuse bleeding and/or post-operative pain. For true reasons, applying tacks in the triangle of doom is forbidden, and using the electrocautery requires increased attention [23–25].

The triangle of pain, located between the superior-lateral iliopubic tract and the inferior-medial gonadal vessels, harbors the femoral nerve, the femoral branch of the genitofemoral nerve, the anterior femoral cutaneous nerve, and the lateral femoral cutaneous nerve. Using tacks in this area may result in chronic postoperative inguinal pain [25].

Corona mortis is an anastomosis between the obturator vessels and the external iliac or branches. Ates *et al*. mention that 30% of patients have some form of corona mortis and are susceptible to vascular injury during the dissection of the Retzius space to Cooper's ligament [26].

### Vascular bundles and nerves

Between the deep inferior epigastric vein, the iliopubic vein, and the communicating rectus-epigastric veins, the so-called Bendavid Venous Circle is formed, which is important in establishing the fixation of the prosthetic material in order to avoid possible post-operative bleeding and other complications that could change the course of the patient's recovery [27]. A correct understanding of the anatomy and vascular bundles leads to a higher quality of surgery and minimizes post-operative complications, especially chronic post-operative pain. Although anatomical variations exist, the main nerves that may alter the patient's post-operative outcome should be known. The lateral cutaneous femoral nerve, originating in the L2 and L3 lumbar branches, has a descending - external path in the iliac fossa and reaches the anterior subcutaneous of the anterosuperior iliac spine. It innervates the anterolateral aspect of the thigh in the middle 1/3[28]. The femoral nerve originates in the L2, L3, and L4 lumbar branches. It innervates the sensory and motor region of the anterior thigh. The genitofemoral nerve originates in the L1 and L2 lumbar branches. This nerve divides the anterior side of the psoas into 2 branches: the genital branch enters the inguinal canal and provides the sensory and motor innervation of the scrotum and cremaster muscle in men, respectively the labia majora in women, and the femoral branch that provides the sensory innervation of the skin of the femoral region (Scarpa's triangle) [29]. The iliohypogastric and ilioinguinal nerves are not as important in the posterior approach and are found in the anterior open approach. [12, 26, 29, 30].

## MATERIAL AND METHODS

The standardized plan that we have been able to develop in the surgical department is based on the follow-up of all the anatomical elements and a wide dissection according to the intraoperative classification of the inguinal hernia. This includes some of the most relevant criteria to develop a safe technique to prevent surgical and post-operative complications (chronic pain, hematoma, seroma) or recurrence.

1. Firstly, a complete examination of the peritoneal cavity is mandatory in any surgery to rule out other conditions that contraindicate the surgical cure of inguinal hernia. After the complete examination, the anatomical landmarks, such as the umbilical ligaments and the fossae of the inguinal region, are identified. It should be noted that peritoneal adhesions should not be dissected unless the inguinal region cannot be visualized.
2. The TAPP procedure involves initiating the peritoneal flap opening at a minimum distance of 4 cm above the inguinal ring, starting from the upper iliac level to the medial umbilical ligament. Careful attention must be given to the epigastric vessels to avoid potential bleeding. Medially, at the level of the medial umbilical ligament, we can encounter the remaining umbilical artery. A minimal incision parallel to the medial umbilical ligament can be practiced to access the deep planes easily. This dissection allows the placement of a prosthetic material of at least 15/10 cm to cover all inguinal (indirect, direct, and femoral) defects, each with about 3-4 cm. With correct dissection, we can also benefit from the least traumatic closure of the peritoneal flap with a resorbable surgical suture or surgical glue.
3. The dissection progresses from the lateral to medial, followed by identifying the iliopsoas muscle and Cooper's ligament within the Bogros and Retzius spaces, respectively. This avascular plane facilitates complete dissection and accurate identification of the fossae after peritoneal dissection. During the procedure, surgeons must be vigilant for small vessels and nerves to ensure post-operative pain control. In order to establish the lower limit of dissection, the pubes should be uncovered, and the dissection should be stopped at about 2 cm below this level. This may also rule out other hernias, such as obturator hernia (located under Cooper's ligament and inferomedial ligament in relation to the femoral sheath).

According to Furtado *et al*. [9], starting the dissection laterally to the medially or medially to the lateral and leaving the middle area to be dissected at the end is recommended. After the dissection of the Retzius space, the most consistent anatomy is given by identifying the pubic bone, allowing better access to the preperitoneal space. The dissection must follow the peritoneal plane, and here in the preperitoneal space, fat tissue can be found, which must be kept close to the abdominal wall and not to the peritoneum. The plane that evidentiates the muscles must be avoided to avoid bleeding from the epigastric vessels. The nerves should not normally be dissected like in the anterior open approach (they allow a better post-operative evolution of the patient by reducing chronic post-operative pain).

An important element in the dissection technic of the medial space is that, at some point, the surgeon must pierce the transversalis fascia to obtain adequate medial exposure, while on the lateral side, the fascia should be intact.

## RESULTS

During the surgical activity performed, including 127 TAPP procedures for inguinal repair, we followed the medial-to-lateral dissection plane with the dissection of the Retzius space and visualization of the pubic bone. Subsequently, lateral dissection of the Bogros space was performed until the iliopsoas muscle was highlighted. It should be noted that no post-operative complications due to bleeding or chronic post-operative pain were observed in the 127 TAPP procedures.

## DISCUSSION

The posterior to anterior dissection in inguinal hernia repair offers several advantages over the anterior approach, enabling better visualization of critical anatomical structures and improving surgical outcomes. One of the notable benefits is the enhanced view of the obturator orifice, which allows for the accurate removal of lipomas at this level. By addressing lipomas more precisely, the procedure can alleviate chronic pain or discomfort in the groin that might persist with the anterior approach.

In order to rule out a femoral hernia, we can mention that the external iliac vein must be visible but not dissected. The possible identification of lymph nodes in the external iliac vein must be very careful not to be considered preperitoneal fat that can be considered a femoral hernia (intraoperative differential diagnosis).

When dissecting indirect hernias, special attention is given to the "indirect triangle." The peritoneum is mobilized to identify the elements of the spermatic cord, such as the spermatic vessels and vas deferens. In female patients, it should be considered that the round ligament of the uterus is attached to the peritoneum. Then resection is indicated at approximately 1 cm proximal to the deep inguinal ring in order not to damage the genital branch of the genitofemoral nerve. There have been no gynecological complications following a hysterectomy, indicating that the procedure can be considered safe [6].

Inadequate parietalization of the spermatic funicular elements is common during the dissection of the indirect sac and peritoneum. To ensure proper compliance, dissection should continue until the elements of the spermatic cord are no longer affected by the traction of the peritoneum. Moreover, the hernial sac should be removed from the transversalis fascia without tearing the spermatic fascia to avoid possible bleeding or injury to the elements of the spermatic funiculus.

In the case of a large indirect hernia, it is recommended to section the hernial sac after the anatomical elements at this level have been identified and removed, leaving the rest of the hernial sac at the level of the scrotum. This maneuver can be considered important for the post-operative evolution of patients, as it can avoid testicular devascularization or possible hematoma at this level, considering that the occurrence of a seroma is much easier to manage in the postoperative recovery [9]. During the dissection, the psoas muscle and iliac vessels can be visualized while gently pulling up the sac and peritoneum to prevent inadvertent movement of the spermatic funicular elements. Dissecting between the elements of the cord helps avoid missing any portion of the sac.

Assessment of the hernial sac and hernia classification are essential steps. Ensuring that the prosthetic material covers the entire myopectinal area facilitates a thorough inspection of the surgical field and the identification of triangles and circles. This classification process prompts surgeons to reassess the local anatomy, contributing to a more accurate and tailored approach to inguinal hernia repair. The completion of parietalization of the spermatic funicular elements is marked by the vas deferens crossing over the external iliac vein and the posterior identification of the iliopsoas muscle.

## CONCLUSION

Continuous improvement in surgical techniques has been instrumental in reducing post-operative complications across various procedures, exemplified by the critical view of safety in laparoscopic cholecystectomy. We believe that CVMO can modify the rate of postoperative complications in the surgical cure of laparoscopic inguinal hernia. For this, a comprehensive understanding of the inguinal region anatomy with posterior to anterior view is needed and the essential areas in performing complete and safe dissection of these spaces. Between March 2019 and March 2023, we performed 127 surgeries with the correct identification of the anatomy and essential spaces of the inguinal region, and the rate of postoperative complications was significantly reduced compared to a dissection that did not follow the established dissection steps. Perseverance in dissection and identification of essential anatomical elements resulted in no complications or a significantly low rate of complications.
